# Characteristics of Older Intimate Partner Violence Cases in Japan

**DOI:** 10.1093/geroni/igaf122.3137

**Published:** 2025-12-31

**Authors:** Asako Katsumata, Kiyoko Fukushima, Noriko Tsukada

**Affiliations:** Japan Lutheran College, Mitaka, Tokyo, Japan; Japan Lutheran College, Mitaka, Tokyo, Japan; Nihon University, Tokyo, Tokyo, Japan

## Abstract

This study examines factors influencing collaboration between Japan’s Community Comprehensive Support Centers (CCSCs) and Domestic Violence (DV) Support Agencies in addressing cases of older Intimate Partner Violence (IPV). Using data from a national survey conducted in November 2022, we analyzed responses from 1,472 Certified Social Workers (CSWs) at randomly selected CCSCs (n = 3,563). Among 406 identified female IPV cases that persisted from younger to older age (excluding missing values), CSWs sought DV Support Agency cooperation in 102 cases (25.8%) but did not in 293 cases (74.2%). Comparative analysis using the χ² test revealed that CSWs were significantly more likely to seek cooperation when victims were physically independent (p < 0.01), had no dementia (p < 0.001), experienced psychological abuse (p < 0.05), and wished to separate from their abuser (p < 0.05). When seeking cooperation, CSWs were more likely to guide victims toward living apart (p < 0.01). Conversely, in cases where cooperation was not sought, CSWs often addressed IPV as a caregiving issue, focusing on reducing caregiver burden when the abuser was also a caregiver (p < 0.01) or instructing the abuser to cease abuse (p < 0.01). These findings suggest that IPV in later life is often reframed as a caregiving issue, potentially hindering appropriate intervention. To better support older IPV survivors, CSWs require specialized training to distinguish IPV from caregiver burden and collaborate effectively with DV Support Agencies.

